# MiRNA-200b level in peripheral blood predicts renal interstitial injury in patients with diabetic nephropathy

**DOI:** 10.5937/jomb0-40379

**Published:** 2023-03-15

**Authors:** Tingfang Chen, Zhenzhen Jiang, Haiying Zhang, Ruifeng Yang, Yan Wu, Yongping Guo

**Affiliations:** 1 Shanghai Sixth Peopležs Hospital Affiliated to Shanghai Jiao Tong University School of Medicine, Department of Nephrology, Shanghai, China

**Keywords:** MiRNA-200b, DN, renal interstitial injury, MiRNA-200b, DN, intersticijalna povreda bubrega

## Abstract

**Background:**

To uncover the diagnostic potential of peripheral blood microRNA-200b (miRNA-200b) in renal interstitial injury in diabetic nephropathy (DN) patients.

**Methods:**

A total of 50 diabetes subjects, 50 mild DN subjects, 50 moderate-severe DN subjects and 50 healthy subjects were included. Peripheral blood level of miRNA-200b in every subject was detected by reverse transcriptase-polymerase chain reaction (RT-PCR). Serum levels of renal function indicators were determined by enzyme-linked immunosorbent assay (ELISA). Meanwhile, relative levels of fibrosis damage indicators were examined by chemiluminescent immunoassay. Diagnostic potentials of miRNA200b in diabetes, mild DN and moderate-severe DN were assessed by depicting receiver operating characteristic (ROC) curves.

**Results:**

Peripheral blood level of miRNA-200b was higher in DN subjects than diabetes subjects without vascular complications, especially moderate-severe DN patients. Peripheral blood level of miRNA-200b in DN subjects was negatively correlated to relative levels of serum creatinine, urinary nitrogen, cystatin, TGF-b, CIV and PCIII. ROC curves demonstrated diagnostic potentials of miRNA-200b in mild and moderate-severe DN.

**Conclusions:**

Peripheral blood level of miRNA-200b is closely linked to the degree of renal interstitial injury in DN patients. MiRNA-200b may be a vital indicator in predicting the development of DN.

## Abstract (sr)

**Uvod:**

Cilj je bio da se otkrije dijagnostički potencijal mikroRNA-200b periferne krvi (miRNA-200b) kod intersticijalne povrede bubrega kod pacijenata sa dijabetičkom nefropatijom (DN).

**Metode:**

Uključeno je ukupno 50 ispitanika sa dijabetesom, 50 ispitanika sa blagim DN, 50 umereno-teškim i 50 zdravih ispitanika. Nivo miRNA-200b u perifernoj krvi kod svakog ispitanika je detektovan lančanom reakcijom reverzne transkriptaze-polimeraze (RT-PCR). Serumski nivoi indikatora bubrežne funkcije određivani su enzimskim imunosorbentnim testom (ELISA). U međuvremenu, relativni nivoi indikatora oštećenja fibroze su ispitani hemiluminiscentnim imunotestom. Dijagnostički potencijali miRNA-200b kod dijabetesa, blage DN i umereno-teške DN procenjeni su prikazom krive operativnih karakteristika prijemnika (ROC).

**Rezultati:**

Nivo miRNA-200b u perifernoj krvi bio je viši kod ispitanika sa DN nego kod ispitanika sa dijabetesom bez vaskularnih komplikacija, posebno kod pacijenata sa umereno-teškim DN. Nivo miRNA-200b u perifernoj krvi kod DN subjekata je bio u negativnoj korelaciji sa relativnim nivoima serumskog kreatinina, azota u urinu, cistatina, TGF-b, CIV i PCIII. ROC krive su pokazale dijagnostičke potencijale miRNA-200b u blagom i srednje teškom DN.

**Zaključak:**

Nivo miRNA-200b u perifernoj krvi je bio usko povezan sa stepenom intersticijalnog oštećenja bubrega kod pacijenata sa DN. MiRNA-200b može biti vitalni indikator u predviđanju razvoja DN.

## Introduction

Diabetic nephropathy (DN) is an important microvascular complication of diabetes, which is the most common cause of end-stage renal failure (ESRD) [1]. It is estimated that by 2045, the number of diabetes patients worldwide will reach 693 million [2]. Sustained hyperglycemia results in extensive vascular damage to eyes, kidneys, heart, and nerves. About 40% of diabetic patients are susceptible to DN [3]. At present, renal biopsy and urine microalbumin detection are the major approaches to diagnose and monitor DN. However, renal biopsy is an invasive examination that is not acceptable to every DN patients and it fails to reflect the severity of DN [4]. It is of significance to develop effective and specific biomarkers of DN.

MicroRNAs (miRNAs) are endogenous, single-stranded RNAs containing 21–25 nucleotides [5]. They are tissue- and time-specific. Through inducing mRNA degradation and blocking protein translation, miRNAs exert post-transcriptional regulations [6]. Functionally, miRNAs are extensively involved in early embryonic development, gene expressions, cell phenotypes, etc. [7] [8]. They also display a certain role in the development of kidney diseases [9]. Detection of miRNA levels in blood or urine may contribute to early screening and disease monitoring of DN.

MiRNA-200 family is a cluster of epithelial-mesenchymal transition (EMT)-associated miRNAs. In particular, miRNA-200b is considered as a negative regulator in tumor metastasis [10]. It is reported that miRNA-200b initiates EMT by interacting with ZEB1/2 [11]. Intercellular TAMs actively participate in tumor neovascularization by regulating EMT and enhancing tumor microvessel density [12]. A relevant study showed that miRNA-200b protects diabetic retinopathy by downregulating VEGFA [13]. Our study aims to uncover the role of miRNA-200b in the development of DN and its diagnostic potential.

## Materials and methods

### Baseline characteristics

This study was performed after obtaining the approval of The Ethic Committee of Shanghai Sixth People's Hospital and the informed consent from the subjects. A total of 50 diabetes subjects without any vascular complications, 50 mild DN subjects (Mogensen II) and 50 moderate-severe DN subjects (Mogensen III-IV) were included. During the same period, 50 healthy subjects undergoing healthy examinations were included. Diabetes and DN were diagnosed based on the standard criteria [14] and kidney biopsy, respectively. Inclusion criteria were: (1) Diagnosis as type 2 diabetes mellitus; (2) DN in Mogensen II-IV; and (3) BMI: 18.5-27 kg/m^2^. Exclusion criteria were: (1) Subjects with urinary calculi, cysts or other occupying lesions; (2) Cerebral infarction; (3) Defect of immune system; (4) Hormone drugs or immunomodulators used in the past 6 months and (5) allergic constitution or history of allergies.

### Blood sample collection

5 mL of venous blood was extracted in each subject under the fasting state in the morning. Blood was centrifuged at 3,000 r/min for 10 min, and the serum was collected and stored at -80°C.

### Reverse transcriptase-polymerase chain reaction (RT-PCR)

TRIzol method (Invitrogen, Carlsbad, CA, USA) was applied for isolating RNAs from serum samples. Through reverse transcription of RNA, the extracted complementary deoxyribose nucleic acid (cDNA) was used for PCR detection by SYBR Green method (TaKaRa, Tokyo, Japan). Primer sequences were listed as follows. MiRNA-200b, F: 5'-GCGGCTAATACTGCCTGGTAA-3', R: 5'-GTGCAGGGTCCGAGGT-3'; and U6, F: 5'-CGCTTCGGCAGCACATATA-3', R: 5'-TTCACGAATTTGCGTGTCAT-3'. The primers were designed based on a previous literature [15].

### Determination of serum markers

Renal function indicators, including serum creatinine, urinary nitrogen, uric acid, and cystatin C were measured through enzyme-linked immunosorbent assay (ELISA) (R&D Systems, Minneapolis, MN, USA) by sarcosine oxidase method, immunoturbidimetry method, enzymatic method and immunoturbidimetry, respectively. Fibrosis damage indicators were examined by chemiluminescent immunoassay.

### Statistical analyses

Statistical Product and Service Solutions (SPSS) 20.0 (IBM, Armonk, NY, USA) was used for all statistical analysis. Data were expressed as mean ± SD (standard deviation). Differences between two groups were analyzed by using the Student's t-test. Comparison between multiple groups was done using One-way ANOVA test followed by Post Hoc Test (Least Significant Difference). Pearson correlation test was conducted for assessing the relationship between miRNA-200b level and serum markers. Receiver operating characteristic (ROC) curves were depicted for evaluating diagnosis potentials of miRNA-200b. P<0.05 indicated the significant difference.

## Results

### Baseline characteristics of subjects

Among 50 healthy subjects, there were 24 males and 26 females, with the age of 32–65 years (mean: 44.18±6.75 years). Their mean BMI and HbA_1c_ were 23.15±3.32 kg/m^2^ and 5.85±0.74%, respectively. Among 50 diabetes subjects, there were 26 males and 24 females, with the age of 34–69 years (mean: 46.23±7.85 years). Their mean BMI and HbA_1c_ were 24.73±3.11 kg/m^2^ and 6.85±0.93%, respectively. Among 50 mild DN subjects, there were 22 males and 28 females, with the age of 31–69 years (mean: 44.91±6.08 years). Their mean BMI and HbA_1c_ were 23.01±3.65 kg/m^2^ and 7.31±0.86%, respectively. Among 50 moderate-severe DN subjects, there were 23 males and 27 females, with the age of 36–60 years (mean: 45.21±5.45 years). Their mean BMI and HbA1c were 23.23±3.24 kg/m^2^ and 7.85±0.93%, respectively. No significant differences in age, gender and BMI were identified among the four groups (Table 1).

**Table 1:** Baseline characteristics of subjects Note: Compared to controls, ^a^P< 0.05; compared to diabetes group, ^b^P<0.05; compared to mild DN group, ^c^P<0.05.

Groups	Age	Sex<br>(male/female)	BMI<br>(kg/m^2^)	HbA_1c_ <br> (%)
Controls	44.18±6.75	24/26	23.15±3.32	5. 85±0.74
Diabetes	46.23±7.85	26/24	24.73±3.11	6. 85±0.93^a^
Mild DN	44.91±6.08	22/28	23.01±3.65	7. 31±0.86_ab_
Moderate-severe DN	45.21±5.45	23/27	23.23±3.24	7. 85±0.93^abc^
F/χ^2^	0.148	0.702	3.216	53.626
P	0.931	0.873	0.024	<0.001

### Peripheral blood level of miRNA-200b

RT-PCR data showed that peripheral blood level of miRNA-200b was higher in healthy subjects than diabetes and DN subjects. In particular, miRNA-200b level was lower in DN subjects than diabetes subjects, especially moderate-severe DN subjects (Table 2). It is indicated that miRNA-200b may be favorable to prevent DN development.

**Table 2:** Peripheral blood level of miR-200b detected by RT-PCR Note: Compared to controls, ^a^P< 0.05; compared to diabetes group, ^b^P<0.05; compared to mild DN group, ^c^P<0.05.

Groups	n	Relative expression<br>of miR-200b
Controls	50	1.885±0.647
Diabetes	50	1.351±0.477^a^
Mild DN	50	0.917±0.328^ab^
Moderate-severe DN	50	0.792±0.204^ab^
F	54.131	
P	<0.001	

### Renal function indicators

Relative levels of serum creatinine, urinary nitrogen, uric acid and cystatin were lower in healthy subjects than diabetes and DN subjects. Notably, the highest levels of renal function indicators were found in moderate-severe DN subjects, followed by mild DN subjects and diabetes subjects (Table 3). We believed that renal function indicators contribute to assess the severity of DN.

**Table 3:** Serum markers of renal function Note: Compared to controls, ^a^P< 0.05; compared to diabetes group, ^b^P<0.05; compared to mild DN group, ^c^P<0.05.

Groups	Serum creatinine<br>(μmol/L)	Urinary nitrogen<br>(mmol/L)	Uric acid<br>(μmol/L)	Cystatin<br>(mg/L)
Controls	60.22±9.33	4.97±0.51	210.22±22.53	0.89±0.14
Diabetes	75.31±11.6^a^	8.16±1.25^a^	258.84±38.62^a^	1.23±0.25^a^
Mild DN	110.5±15.21^ab^	12.83±1.96^ab^	345.94±45.17^ab^	1.56±0.30^ab^
Moderate-severe DN	152.15±20.62^abc^	15.62±2.13^abc^	489.25±56.38^abc^	1.81±0.42^abc^
F	570.01	523.098	426.361	101.1
P	<0.001	<0.001	<0.001	<0.001

### Serum markers of fibrosis damage

Serum markers of fibrosis damage, including TGF-β, HA, CIV and PCIII were examined in each subject. Relative levels of fibrosis damage indicators were lower in healthy subjects than diabetes and DN subjects. The highest levels were seen in moderate-severe DN subjects (Table 4). Therefore, serum markers of fibrosis damage may also be used to assess the severity of DN.

**Table 4:** Serum markers of fibrosis damage Note: Compared to controls, ^a^P< 0.05; compared to diabetes group, ^b^P<0.05; compared to mild DN group, ^c^P<0.05.

Groups	TGF-β (μg/L)	HA (μg/L)	CIV (μg/L)	PCIII (μg/L)
Controls	5.28±0.8	30.85±4.69	41.29±6.33	23.69±2.69
Diabetes	8.25±0.91^a^	42.68±5.24^a^	59.14±6.85^a^	32.75±3.22^a^
Mild DN	12.24±1.36^ab^	58.33±6.96^ab^	67.32±7.17^ab^	44.23±4.16^ab^
Moderate-severe DN	15.21±1.98^abc^	78.91±9.51^abc^	89.53±9.24^abc^	55.98±5.96^abc^
F	621.003	524.517	280.321	646.206
P	<0.001	<0.001	<0.001	<0.001

### Pearson correlation test on miRNA-200b level and serum markers

We have proven that relative levels of miRNA-200b, renal function indicators, and serum markers of fibrosis damage were different in diabetes and DN subjects. Subsequently, Pearson correlation test showed that peripheral blood level of miRNA-200b was negatively correlated to serum creatinine, urinary nitrogen, cystatin, TGF-β, CIV and PCIII (r = -0.521, -0.683, -0.683, -0.811, -0.588 and -0.721, respectively) in DN subjects (Table 5).

**Table 5:** Pearson correlation test on miRNA-200b level and serum biomarkers. *P<0.05

Serum marker	Controls		Diabetes		DN	
	r	P	r	P	r	P
Serum creatinine	-0.256	0.095	-0.512	0.064	-0.521	0.002*
Urinary nitrogen	-0.239	0.06	-0.115	0.157	-0.683	0.018*
Uric acid	-0.125	0.335	-0.442	0.095	-0.522	0.071
Cystatin	-0.557	0.497	-0.109	0.415	-0.683	0.029*
TGF-β	0.467	0.082	-0.254	0.155	-0.811	<0.001*
HA	-0.425	0.466	-0.328	0.261	-0.462	0.627
^C^IV	-0.267	0.185	-0.612	0.32	-0.588	0.026*
^PC^III	0.359	0.447	-0.324	0.054	-0.721	0.005*
HBA1c	-0.305	0.064	-0.287	0.981	-0.253	0.143

### Diagnostic potentials of miRNA-200b in DN

**Figure 1:**
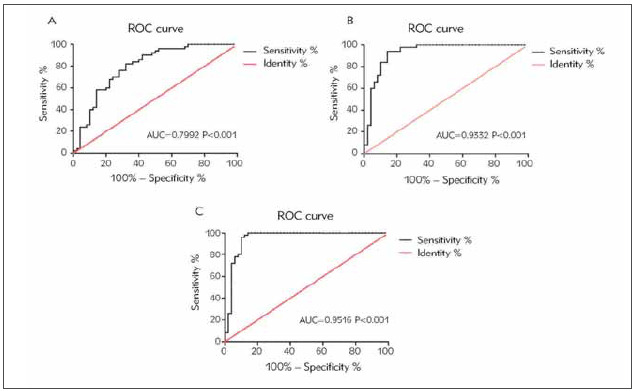
Diagnostic potentials of miRNA-200b in diabetes, mild DN and moderate-severe DN. (A) Diagnostic potential of miRNA-200b in diabetes (AUC=0.7992, P<0.001); (B) Diagnostic potential of miRNA-200b in mild DN (AUC=0.9332, P<0.001); (C) Diagnostic potential of miRNA-200b in moderate-severe DN (AUC=0.9516, P<0.001)

ROC curves were depicted for assessing diagnostic potentials of miRNA-200b in DN. Sensitivity and specificity of miRNA-200b in diagnosing diabetes were 76% and 72%, respectively (AUC=0.7992, *p*<0.001, cut-off value=1.636) (Figure 1A). MiRNA-200b was able to diagnose mild DN (sensitivity=90%, specificity=86%, AUC=0.9332, P<0.001, cut-off value=1.294) (Figure 1B). Sensitivity and specificity of miRNA-200b in diagnosing moderate-severe DN were 88% and 90%, respectively (AUC=0.9516, P<0.001, cut-off value=1.092) (Figure 1C). It is concluded that miRNA-200b was able to diagnose diabetes, mild DN and moderate-severe DN.

## Discussion

It is estimated that by 2030, 7.7% of people aging 20–79 years suffer from diabetes [16]. DN is a severe complication of diabetes. Uncontrolled DN will deteriorate into ESRD that is difficult to be treated. Current therapeutic strategies of DN aim to control blood glucose, blood pressure and lipids [17]. Nevertheless, the development of DN cannot be reversed or blocked. Prevention and intervention of DN in the early stage are of significance.

A single miRNA can bind several target genes, thereafter influencing gene expressions and functions [18]. Differentially expressed miRNAs in kidney tissues of DN patients are able to reflect the disease condition [19]. MiRNAs are stably expressed in serum, and detection of serum miRNAs is sensitive and specific [20]. It is reported that miRNAs are involved in thickening of the glomerular basement membrane, podocyte apoptosis, deposition of extracellular matrix, cell fibrosis, etc., and eventually lead to the development of DN [21]. MiRNAs are believed as promising biomarkers in diagnosis and monitoring of DN. Bai et al. [22] proposed that miRNA-130b is downregulated in kidney tissues of DN patients. MiRNA-130b alleviates EMT-induced fibrosis in rat renal tubular epithelial cells through downregulating Snail. A prospective study conducted in Europe involving 455 type 1 diabetes mellitus patients uncovered that serum level of miRNA-126 is negatively linked to susceptibilities to diabetic vascular complications, especially proliferative kidney diseases [23].

In this trial, we found out that miRNA-200b level was downregulated in peripheral blood of DN subjects, especially moderate-severe DN subjects. Subsequently, potential relationship between miRNA-200b level and renal function and fibrosis damage indicators was analyzed. Pearson correlation test showed that peripheral blood level of miRNA-200b was negatively correlated to serum creatinine, urinary nitrogen, cystatin, TGF-β, CIV and PCIII in DN patients. Such a correlation was not identified in diabetes patients without vascular complications, suggesting that renal function may be normal in diabetes patients. ROC curves analyses further demonstrated the diagnostic potentials of miRNA-200b in mild and moderate-severe DN. However, there are still two shortcomings in this study. Firstly, evaluation indicators of renal interstitial fibrosis lack organ specificity. Secondly, the role of miRNA-200b may be varied in DN with different pathological stages. Our results should be validated in future explorations.

Several previous studies demonstrated that miR-200 family may be involved in the development of diabetic nephropathy, but most of these studies focused on molecular mechanisms rather than directly analyzing clinical data through peripheral blood samples of patients [24] [25]. Compared with previous studies, the most significant innovation of this study is that it is the first study to focus on the expression level of miR-200b in peripheral blood of patients with diabetic nephropathy and its clinical value.

## Conclusions

Peripheral blood level of miRNA-200b is closely linked to the degree of renal interstitial injury in DN patients. MiRNA-200b may be a vital indicator in predicting the development of DN.

## Dodatak

### Financial Disclosure

The authors declared that this study has received no financial support.

### Conflict of interest statement

All the authors declare that they have no conflict of interest in this work.
